# Improving Community Health Worker Compensation: A Case Study From India Using Quantitative Projection Modeling and Incentive Design Principles

**DOI:** 10.9745/GHSP-D-21-00413

**Published:** 2022-06-29

**Authors:** Mokshada Jain, Yael Caplan, Banadakoppa Manjappa Ramesh, Hannah Kemp, Bettina Hammer, Shajy Isac, James Blanchard, Vasanthakumar Namasivayam, Sema K. Sgaier

**Affiliations:** a Surgo Ventures, Washington, DC, USA.; b Department of Community Health Sciences, University of Manitoba, Winnipeg, Canada.; c India Health Action Trust, Lucknow, India.; d Department of Global Health & Population, Harvard T.H. Chan School of Public Health, Boston, MA, USA.; e Department of Global Health, University of Washington, Seattle, WA, USA.

## Abstract

**Introduction:**

Although community health workers (CHWs) are effective at mobilizing important health behaviors, there is limited evidence on how financial incentive systems can best be designed to drive their effectiveness. This study intends to bridge this evidence gap by analyzing the compensation model of India's accredited social health activist (ASHA) program and identifying areas of improvement in the system's design and implementation.

**Methods:**

We analyze the ASHA program in Uttar Pradesh, India. ASHAs receive compensation through a mix of program-linked, performance-based, and routine activity-based incentive structures. Using multiple data sources, including a novel linked household and ASHA survey, we estimate ASHA performance-linked incentive earnings under different scenarios of ASHA actions and household behaviors. Juxtaposing statistical projection models and actual government payments, we identified which incentives promised the highest payments, which were claimed or not, which could be claimed more by increasing ASHA actions, and which were paid despite not meeting payment criteria. We also report findings on ASHA awareness of and experiences with claiming incentives.

**Results:**

We find crucial gaps and implementation challenges in the ASHA incentive structure. ASHAs could double their earnings by completing certain tasks within their control. ASHAs may also be paid for partial completion of activities, as incentives are paid in lump sums for a series of activities rather than for each activity. Family planning incentives have the largest gap between potential and actual earnings. Incentivizing ASHAs for achieving certain health outcomes is inefficient, as no clear linkage was found between the achievability of such health outcomes and the claim amounts.

**Conclusion:**

There are several opportunities for improving CHW compensation, from improving the incentive claims process to shifting focus to achievable outcomes. Optimizing incentive system designs can further enhance CHW effectiveness globally to affect key health behaviors.

## Abstract (precis)

We quantitatively assess the design and performance of the accredited social health activist (ASHA) incentive structure and suggest recommendations that could potentially drive ASHA effectiveness and support the achievement of health outcomes.

## Teaser

**Key Findings:**

- Using earnings projection models, we identified gaps and implementation challenges in the accredited social health activist (ASHA) incentive structure that may affect ASHAs' motivation and desired health outcomes.
- ASHAs could more than double earnings by achieving full coverage and completing activities fully within their control.
- Government payments are higher than the modeled actual earnings, hinting at overpayment due to receipt of full payment despite only partial completion of antenatal care, postnatal care, and immunization incentives and suggesting that the current design does not motivate ASHAs to complete a series of outcomes.

**Key Implications:**

- Challenges such as issues in the disbursement of payments, payment on partial completion of tasks, incentive dependence on household actions, ASHAs not performing at their full potential, and lacking awareness of the quantum of incentive present several opportunities for policy makers to improve CHW compensation structures. Possible courses of action include improving the incentives model design to help community health workers achieve outcomes and the incentive tracking and payments machinery.
- Updating incentive structures for community health workers using lessons on incentive design from the field can improve CHW effectiveness in India and globally.

## INTRODUCTION

Globally, community health workers (CHWs) deliver and connect people to essential health services. There are more than 5 million CHWs in over 20 countries, from Brazil's Community Health Agents to Bangladesh's Shasthya Shebikas.^1^ CHWs play an important role in influencing many health outcomes including preventing undernutrition, reducing neonatal and child mortality, managing childhood illness, and HIV prevention.^1^^,^^2^

Despite the recognized importance of CHWs, there are many knowledge gaps around how to improve their effectiveness and role.^3^ One important question is what compensation should be offered to CHWs to motivate them and drive program objectives. There is a growing consensus, including by the World Health Organization, that CHWs should receive financial compensation commensurate with their effort and skills.^4^^–^^6^ However, different compensation models exist globally, from salaries to performance-based financial incentive models,^3^^,^^7^^,^^8^ and reviews of programs in low- and middle-income countries yield mixed results for the relationship between the compensation program model and its overall effectiveness.^8^^–^^11^

There is a growing consensus that CHWs should receive financial compensation commensurate with their effort and skills.

For practitioners, it is important to understand whether CHW underperformance stems from the program not being implemented properly or the program design not being optimized for maximum effect.^12^^,^^13^ This is particularly the case for performance-based incentive models, which have come under criticism because they seem to focus CHW attention on those tasks with the greatest incentive amounts at the expense of other tasks.^12^^,^^14^^,^^15^

There is a great need for quantitative evidence on the design and implementation of compensation structures. In this article, we bridge the evidence gap by analyzing the compensation program of one of the world's largest CHW cadres: India's accredited social health activist (ASHA) program, identifying key areas of improvement in the system's design and implementation.

## INDIA'S ASHA PROGRAM

Trained volunteer female CHWs called ASHAs play a large role in health care promotion in rural India. In 2005, the ASHA program was established as part of the Indian Government's National Rural Health Mission.^16^ ASHAs are selected by local village committees according to national guidelines. They receive 23 days of training over the course of a year, the specific content of which may vary by state. ASHAs are supervised by an ASHA facilitator who oversees a group of approximately 20 ASHAs. The supervisor offers mentoring and regular meetings.^17^ ASHAs live in the community and on average serve around 1,000 people. The Government of India relies on ASHAs to mobilize families to seek and access appropriate care, provide counseling on healthy behaviors, distribute drugs and health commodities, provide monitoring/screening services, and be a health activist who raises her community's health awareness.^18^ The national government determines the overall structure of the ASHA role, but the program details vary from state to state. Currently, most of the ASHA's work revolves around reproductive, maternal, neonatal, and child health (RMNCH), although other areas, such as noncommunicable diseases, are continually added to her portfolio. This is true in Uttar Pradesh, India's most populous state with a population of more than 200 million, where approximately 155,000 ASHAs have an ever-expanding portfolio of duties.^19^ The ASHA program has been effective at supporting maternal and child health across India: ASHA services have been shown to be associated with increased uptake of antenatal care (ANC) visits, skilled birth attendance, and facility delivery.^17^

Although ASHAs are volunteers, they receive compensation through a mix of program-linked, performance-based, and routine activity-based incentive structures. ASHAs reported having a renewed sense of identity, feeling more self-confident, being less financially dependent on their spouse and family, sharing the household work burden with family, and having an increased say in decisions concerning household management and seeking health care. Although incentives provide the system with a mechanism to hold ASHAs accountable for their performance, it also makes some in the ASHA's community skeptical of her motives. The ASHA incentive structure is complex with a national model which states can adapt. ASHAs generally can receive over 40 different incentives for a variety of tasks, with new incentives occasionally added. Examples of performance-based incentives in Uttar Pradesh and many other states include INR 300 (US$4) for assisting with an institutional delivery and INR 100 (US$1) for performing complete immunization of a child in the first year.^20^ Since 2016, in addition to performance-based incentives, incentives are provided for a set of routine and recurring activities around conducting and attending meetings and undertaking village census to regularly maintain their beneficiary lists.^21^ A key difference between program-linked, performance-based, and routine activity-based incentives is that the earnings from the latter are fixed regardless of the size of the ASHA catchment area.

Incentives are awarded based on a mix of ASHA effort and ability to persuade individuals as well as factors outside their control. Some of the incentives depend only on ASHAs completing routine tasks, such as attending monthly meetings, or providing services to households, such as completing postnatal care home visits. In contrast, payment for other incentives is contingent on ASHAs being able to influence households so that they perform certain health behaviors, such as adopting a permanent contraception method after 2 children or delivering at a public health facility. Further, some incentives are given for the completion of one-time outcomes, such as delivering a child at a facility or getting sterilized, whereas others are for the completion of a series of activities, such as a woman attending 4 ANC check-ups or ASHAs making 6–7 home visits to a recently delivered woman and infant. A key question that arises: Is this incentive system optimally designed to achieve the desired health outcomes for the households ASHAs serve? Given that ASHAs are effective at mobilizing women to seek care, further optimization of the incentive system can have important community health benefits.

Is the current incentive system optimally designed to achieve the desired health outcomes for the households ASHAs serve?

Research on the ASHA program suggests that the financial remuneration via incentives is a key motivator for ASHAs and that they focus their time on incentivized tasks.^5^^,^^8^^,^^13^^,^^14^ After the implementation of incentives for institutional delivery for both ASHAs and households, many states in India saw a large increase in institutional delivery rates.^22^^,^^23^ ASHAs report being motivated by financial incentives and satisfied by their ability to earn and contribute to their households.^13^^,^^24^ CHWs from other parts of the world also appreciate and welcome financial incentives in the form of salaries or allowances.^4^

But financial incentives are not the only motivator for CHWs, and there is heterogeneity in CHW, including ASHA, preferences.^4^^,^^5^^,^^24^^,^^25^ Existing literature suggests that CHWs across countries are driven by intrinsic motivational factors. Some even consider nonmaterial incentives such as community recognition, professional development, and peer relations more important than financial incentives.^4^^,^^26^ While nonfinancial performance-based incentives like supportive supervision, adequate resources, and community respect can increase CHW service delivery outcomes, when these are absent or when incentive expectations go unmet, they can lead to demotivation.^4^^,^^5^^,^^14^^,^^24^^,^^26^^,^^27^ Suboptimal program design and implementation can also constrain the motivation ASHAs derive from incentives.^13^ Challenges such as delayed and irregular payment disbursement, incentives linked to client behavior, and unfair distribution of incentives can influence job motivation and satisfaction of CHWs in India and other low- and middle-income countries.^4^^,^^5^^,^^15^

Qualitative research with ASHAs has uncovered an array of complaints about the incentive system's design and implementation. Incentive payments to ASHAs can be limited, inconsistent, and irregular.^13^^–^^15^^,^^24^ One review of the ASHA incentive program found that while it has successfully implemented guidelines, regular communications, and strong management, it has weaknesses including delays and lack of clarity around payment.^28^ Documenting task completion can be burdensome, particularly for ASHAs with low literacy. Moreover, ASHAs lack awareness of which tasks have associated incentives and their incentive amounts.^15^^,^^18^ Some incentives can only be achieved conditional on the patient's behavior, which is not fully within the ASHA's control.^4^^,^^5^^,^^13^^,^^15^^,^^24^ These shortcomings in program design and implementation can trigger dissatisfaction, lower motivation, and decrease effort.^4^^,^^13^ For example, due to the challenges in getting paid, some ASHAs find a second job that is more economically rewarding, which limits the time they spend serving the health needs of their community.^14^ These findings indicate that the incentive program is important for driving outcomes, but that shortcomings in design and implementation are limiting its efficacy. However, current research fails to take a holistic approach to demonstrate specific ways the incentive structure can be improved to help ASHAs achieve outcomes.

In this article, we ask: is the incentive system designed and implemented to reward outcomes and motivate ASHAs to complete the activities expected of them? We project ASHA incentive earnings under different scenarios of ASHA actions and household behaviors using a rich array of data sources from Uttar Pradesh, India, including a unique set of linked household and ASHA surveys, and compare results to actual payments for each incentive.^29^ We provide a set of recommendations for improvements to the incentive system and capture more general learnings that can be applied both for ASHAs in India and CHW programs worldwide.

We analyze whether the incentive system is designed and implemented to reward outcomes and motivate ASHAs to complete the activities expected of them.

## METHODS

### Data Sources

Multiple data sources were used for our analysis, including the Surgo-Uttar Pradesh Technical Support Unit (UPTSU) RMNCH survey, which extensively surveyed ASHAs and the households in their catchment areas. We tried to leverage high-quality data sets that were largely statistically robust population-level representative surveys. Supplement Table 1 lists the specific data sets used for each incentive.

#### The Surgo-UPTSU RMNCH Survey With Households and ASHAs

We used the Surgo-UPTSU RMNCH Survey, a linked, population-based survey data set of households (mothers) and ASHAs conducted in Uttar Pradesh (rural) in 2017–2018.^29^ This data set served as the primary source for calculating beneficiary incidence (e.g., number of pregnant women and number of children age 5 years and younger in an ASHA catchment area), ASHA action (e.g., home visits by ASHAs), and household behavior rates (e.g., institutional delivery rate) needed to model ASHA incentive claims. The Supplement includes details of the survey content and variables computed from this data.

#### Other Data Sets

Government health statistics reports were used to compute the incidence rates of pregnant women, newborns, children, and eligible family planning (FP) couples required for incentives concerning ANC, institutional deliveries, postnatal visits, visits to malnourished/underweight children, immunizations, and certain FP incentives respectively. For estimates on crude birth rate and infant mortality rate for rural Uttar Pradesh, we used the Sample Registration System bulletin report released in September 2017.^30^ For neonatal mortality rate estimates, we used the Sample Registration System statistical report 2016.^31^ The 2011 Census data for Uttar Pradesh was used to project and estimate the proportion of children aged 5 years and younger in the population for 2016. A 2012 Government of India Order on the Home Delivery of Contraceptives was used for the eligible couple rate.^32^ We obtained the severely wasted rate of children aged 5 years and younger from the Rapid Survey of Children, 2013–2014.^33^ For behavior rates on child immunizations, we used the National Family Health Survey 2015–2016 state factsheet for rural Uttar Pradesh.^34^

The UPTSU, implemented by India Health Action Trust and embedded in the Government of Uttar Pradesh, is tasked with supporting the state government to increase the efficiency, effectiveness, and equity of the delivery of key RMNCH services. In 2016, the UPTSU administered the District Level Family Planning Survey in 25 high-priority districts in Uttar Pradesh (Supplement). We used District Level Family Planning Survey data on sterilization preference and adoption rates; proportion rates of couples with 0, 1, and 2 children; unmet need; birth spacing; and receipt of counseling by ASHA on limiting and spacing methods from the survey for the FP incentives' earning potential.

We used the UPTSU Community Behavior Tracking Survey data (April–July 2016) to compute the proportion of malnourished children that go to the Nutritional Rehabilitation Centre and the proportion of ASHA home visits received for relevant incentives (Supplement).^35^ To compute the proportion of newborns required to be admitted to a sick newborn care unit for the related incentives, we used the UPTSU case sheet summary data as of August 2016. UPTSU program monitoring data (January 2015–May 2016) was used for data on the proportion ASHAs attending block meetings for the block meeting incentive.

To compare the results of modeled ASHA claims with actual payments, we used the Government of Uttar Pradesh's state-level incentive payments data for the financial year 2017–2018. For FP, excluding postpartum intrauterine contraceptive devices (PPIUCD) and immunization incentives, we used payments data for the financial year 2015–2016 to match the time period of the data used in the earnings projection models for these indicators.

All other rates required for modeling what ASHAs claim were calculated using the Surgo-UPTSU RMNCH survey with households and ASHAs data.

#### Patient and Public Involvement

Those interviewed in the surveys were not directly involved in its design, conduct, analysis, or dissemination.

#### Data Availability

De-identified data can be made available upon request. Reuse is permitted on a case-by-case basis for academic purposes.

### Data Analyses

#### Incentive Claims by ASHAs

To estimate ASHA incentive earnings under different scenarios of ASHA actions and household behaviors, we developed 4 earning projection models (Table). By comparing these models with actual government payments, we were able to understand which incentives promise the highest payments, which incentives are being claimed or not, which incentives can be claimed more by increasing ASHA actions, and which incentives are paid despite not meeting the criteria for payment. We restricted the analysis to 23 RMNCH services and related incentives that constitute the majority of total ASHA earnings as of FY 2017–2018 and classified them into 5 categories: (1) antenatal care and institutional delivery (ANC and institutional delivery), including attend ANC checkups and deliver in a public facility; (2) newborn and child health (PNC), including postnatal care home visits, visiting critically sick newborns, and underweight children; (3) FP, including sterilization, PPIUCD, and birth spacing, (4) routine immunization; and (5) routine activities, including household survey, listing suitable couples and other recordkeeping, and attending meetings. Of the 23 incentives, 19 use statically robust and population-level representative surveys. Owing to the lack of population-level data on sick newborn care unit admissions and community meetings attended by ASHAs, we use service statistics-based data sets for 4 incentives. The data used in the earning projection models do not rely on self-reported actions by the ASHA. The data sets use ASHA actions as reported received by the households and via an independent audit of ASHA recordkeeping by trained research teams for recordkeeping based on routine activities incentives. Supplement Table 2 lists the 23 incentives and details on the data sources used in the earnings projection models.

**TABLE.** ASHA Earning Projection Models, Descriptions, and Use Cases

Model	Description	Use Case	Example Estimation Calculations
Perfect ASHA–perfect household	ASHA completes all her actions, and all households respond perfectly by completing all right behaviors for all incentives.	Maximum possible earnings for an ASHA	To illustrate, for the institutional delivery incentive, we estimated the beneficiary incidence of pregnant women in a population of 1,000, using the crude birth rate in Uttar Pradesh. We arrived at an incidence of 2.3 pregnant women per month and multiplied it by the incentive amount of INR 300 per pregnant woman delivering at a public health facility, to arrive at maximum earnings of INR 682, per month from this incentive. Several incentives only depend on ASHA actions, such as ASHAs updating records or conducting meetings monthly. The incidence of such incentives in the perfect ASHA–perfect household model, in a given month, is 1, which is multiplied by the incentive amount to get the maximum earnings.
Perfect ASHA–actual household	ASHA completes all her actions for all households and individual activities, but the households respond at their “actual” rates given ASHA action (e.g., percentage of mothers who deliver in hospitals receiving ASHA counseling on hospital delivery).	ASHAs' earning potential if they undertake all actions fully under their control	Continuing with the institutional delivery incentive example, the household behavior is a pregnant woman delivering at a public health facility and the ASHA action mapped is ASHA counseling a pregnant woman to opt for facility delivery. In this model, since the ASHA is “perfect,” she will counsel all 2.3 pregnant women, but only a proportion of those women will opt for facility delivery as the households are not “perfect.” From our household survey data, we computed the public facility delivery rate for a pregnant woman that receives counseling on facility delivery from ASHA to be 0.66. We multiplied this rate by 2.3 pregnant women to arrive at an incidence of 1.5 for this model, which was further multiplied with the incentive amount of INR 300 per delivery to arrive at an earning of INR 457 per month for this incentive in the perfect ASHA–actual household model. For incentives such as conducting monthly meetings, which are independent of household behavior, the same earning as the perfect ASHA–perfect household model, is estimated in this model, given the ASHA continues to be “perfect.”
Actual ASHA– perfect household	ASHA partially completes actions at “actual” rates covering only a proportion of households, who respond “perfectly” by completing the right behaviors.	Keeping household coverage at existing levels and improving quality within achieved coverage	To compute the incidence in this model, we multiply the 2.3 pregnant women by 0.6, the proportion of women that receive counseling by ASHAs on hospital delivery. We further multiply the incidence, 1.36, with INR 300 to arrive at INR 409.16 earnings from this incentive in this model. Similarly, for the incentive for monthly recordkeeping of births and deaths, the ASHA receives INR 100 per month. We multiplied the monthly incidence of 1 by 0.75, which is the proportion of ASHAs that maintain this record to arrive at INR 75 for this incentive in this model.
Actual ASHA–actual household	The estimated earnings that an average ASHA should earn given “actual” completion rates of actions by ASHAs and behaviors by households.	Compared with actual payments made by the government to assess the implementation of the incentive system	To obtain the incidence for the institutional delivery incentive in this model, we take the incidence of the previous model of 1.36, which is the number of pregnant women that the ASHA covers to counsel on institutional delivery, and multiply it by 0.66, the facility delivery rate for a pregnant woman that receives counseling by an ASHA, to get an incidence of 0.9. This is multiplied by INR 300 to get an institutional delivery incentive earning of INR 270 in this model. We cannot simply take the rate of public facility delivery in the population as there are women that go for public facility delivery despite no contact with ASHA and in such cases, the ASHA cannot claim her incentive. For incentives not dependent on household behavior, the same earnings are estimated as the actual ASHA–perfect household model.

Abbreviations: ASHA, accredited social health activist; INR, Indian rupee.

To understand the earnings potential associated with each incentive, we categorized relevant ASHA action(s) and household behavior(s) into 2 cases: “perfect” and “actual.” In the “perfect” ASHA case, she completes her actions for all households in her area. It may be the case that households might be exercising a different choice to meet their health goals. In the “perfect” household case, all households in an ASHA area complete the right behavior.

The “perfect” household behavior for the purpose of the incentives model estimations is strictly the clinically recommended behavior by the health system for which ASHAs are given incentives. For example, consider the incentive for encouraging couples to go for permanent contraception method after 2 children. The household might instead choose to use a temporary method of contraception to control birth, but ASHAs will not receive the incentive amount in this case. So, the term “perfect” is strictly used within the scope of the behavior recommended by the health system as laid out in the incentives.

In the “actual” ASHA case, she completes her actions for a proportion of the households in her area or completes actions with an average probability as per actual rates from survey data. For “actual” households, a proportion of households complete the right behavior as per actual rates.

A cross-tabulation of the 4 cases “perfect” and “actual” for ASHAs and households produces 4 possible scenarios represented by 4 models. For each incentive, we mapped each of the relevant ASHA action(s) and household behavior(s). The models' output is estimated monthly ASHA earnings for an average population of 1,000 per ASHA area, for each of the 4 modeled scenarios (Table).

The following assumptions have been made in this analysis. We have assumed an ASHA catchment size of 1,000 population. ASHA catchment sizes can vary quite substantially. Supplement Table 3 shows model results with varying catchment sizes. State-level or grouped priority blocks or district-level averages from different surveys have been used to model all “actual” models (e.g., the average rates of activity completion among ASHAs are used to model the “actual ASHA” and the average rates of behavior uptake among households are used to model the “actual household”) to estimate the average ASHA earnings. These rates vary at the ASHA level, and therefore, the estimated earnings will differ at the individual ASHA level. We assume there are no reporting errors in the state-level government ASHA payment data for the purposes of this analysis.

#### ASHA Incentive Awareness and Experiences

Descriptive analysis was conducted with the Surgo-UPTSU RMNCH survey with households and ASHAs to understand awareness levels and delays in payment.

### Ethics Approval

The studies were approved by the Sigma Institutional Review Board (approval number 10015/IRB/15-16, New Delhi, India) and the University of Manitoba Health Research Ethics Board (approval number HS20187 [H2016:385]). All subjects included in the analyses gave consent.

## RESULTS

### ASHAs Have the Potential to Increase Earnings but Are Paid for Partial Fulfillment of Many Incentives

**FIGURE 1:**
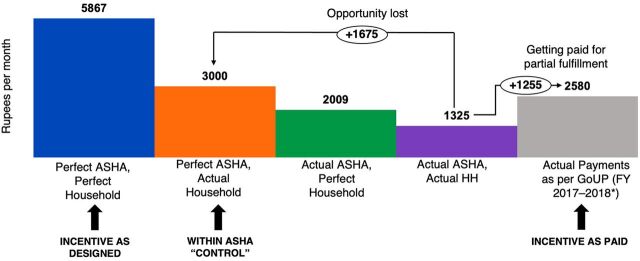
ASHA Earnings Fall Short of Earning Potential^a^ Abbreviations: ASHA, accredited social health activist; FP, family planning; GoUP, Government of Uttar Pradesh. ^a^ There is a large gap between ASHA earning potential, demonstrated by the perfect ASHA-actual household model, and actual earnings, demonstrated by the actual ASHA-actual household model. This is coupled by overpayment, evidenced by the Indian rupee 1,255 difference between the actual ASHA-actual household model and the actual payments reported by the government. State-wide fiscal year 2017–2018 payments data were used except for FP (excluding postpartum intrauterine device) and full immunization, for which fiscal year 2015–2016 data are used given estimates are from 2015–2016 surveys for these indicators.

Results from the earnings projection models and actual payments point to some of the inefficiencies in the incentive design and implementation. We find that in the perfect ASHA–perfect household model where an ASHA completes all her actions and all households respond perfectly by performing the right behaviors, the ASHAs would earn INR 5,867 (US$78) per month. When an ASHA completed all actions under her control and households responded at their actual rates (perfect ASHA–actual household model), ASHAs should earn INR 3,000 (US$40) per month. Modeled earnings given actual completion rates by ASHAs and households were INR 1,325 (US$19) per month (actual ASHA–actual household model). This means there is a lost opportunity of INR 1,675 (US$22): ASHAs could more than double earnings by achieving full coverage and completing activities fully within their control. However, we found that government payments are INR 2,580 (US$36) per month on average, which is INR 1,255 (US$18) higher than the modeled actual earnings, suggesting overpayment (Figure 1).

**FIGURE 2:**
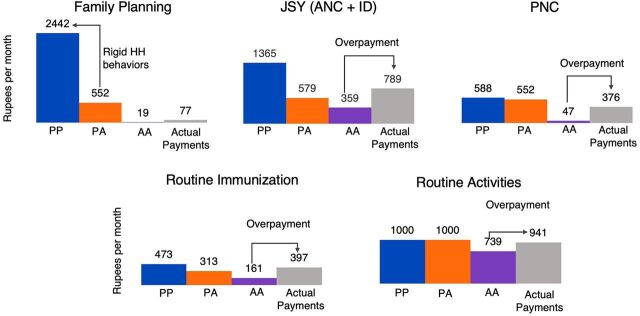
ASHAs Are Overpaid for Many Incentives^a^ Abbreviations: AA, actual ASHA-actual household model; ANC + ID, antenatal care and institutional delivery; ASHA, accredited social health activist; FP, family planning; JSY, Janani Suraksha Yojana safe motherhood intervention; PA, perfect ASHA-actual household model; PNC, postnatal care; PP, perfect ASHA-perfect household model. ^a^ PPIUCD incentive used statewide 2017–2018 FP payment data. For other FP incentives, 2015–2016 HPD data is used to match the 2015–2016 estimates. Payment data for sterilization is not available. So, the average earnings on sterilization as reported by ASHAs have been added. VHND mobilization payment updated with statewide fiscal year 2017–2018 but given immunization data uses National Family Health Survey 2015–2016 data, statewide fiscal year 2015–2016 data is used.

Overpayment is noted particularly for the ANC and institutional delivery incentive, the PNC incentive, and the routine immunization incentive. For ANC and institutional delivery, there was a calculated overpayment of INR 430 (US$6), for PNC an overpayment of INR 329 (US$5), and for routine immunization an overpayment of INR 236 (US$3) (Figure 2). Overpayment is mainly due to receipt of full payment despite only partial completion of ANC, PNC, and routine immunization incentives. ANC, PNC, and immunization incentives require completion of a series of activities/outcomes, but there is a single lump sum incentive amount awarded for the full set. For example, the PNC incentive requires the ASHA complete 6 to 7 visits to the woman after birth.

Overpayment is noted particularly for the ANC and institutional delivery incentive, the PNC incentive, and the routine immunization incentive.

We were able to closely match real payment data by modeling expected payment if the task completion threshold to receive certain incentives was relaxed. For the ANC and institutional delivery incentive, conditions were relaxed to include 1+ ANC checkups only instead of 4+ checkups and 1+ ASHA home visits. This revised model yielded an expected payment of INR 765 (US$11), which was closer to the actual payment of INR 789 for the incentive. For PNC incentives, the conditions of the model were relaxed to include 2+ PNC visits instead of 6 to 7. This model yielded an expected payment of INR 359 (US$5), again closer to the actual payment of INR 376. For routine immunization incentives, if the conditions are relaxed to reduce the number of vaccinations and full coverage for mobilization, the revised model yielded an expected payment of INR 373 (US$5) which closely matched the actual payment of INR 397. These findings indicating payment for partial fulfillment of incentives explain our observed overpayment.

We also identified activities with the largest gaps between potential and actual earnings. Family planning incentives are designed to yield maximum earnings (perfect ASHA–perfect household model), accounting for 42% of potential total earnings, followed by ANC and institutional delivery at 23% and routine activities at 17%. However, our model suggests only 3% of earnings is currently from FP. Instead, most ASHAs earn the bulk of their income from routine activity (36%) and ANC and institutional delivery incentives (31%). The gap in potential and actual earnings for FP can be attributed to rigid household behaviors. There is a large drop-off in earnings between the perfect ASHA–perfect household model earnings and the perfect ASHA–actual household model earnings of INR 1,890. This demonstrates that even if ASHAs did everything under their control for the FP incentive, they would earn far short of the potential due to household behaviors. This demonstrates gaps between the incentive system as designed and the system as implemented.

### ASHAs Show Low Awareness of Exact Incentive Amounts and Report Difficulty Getting Paid

**FIGURE 3:**
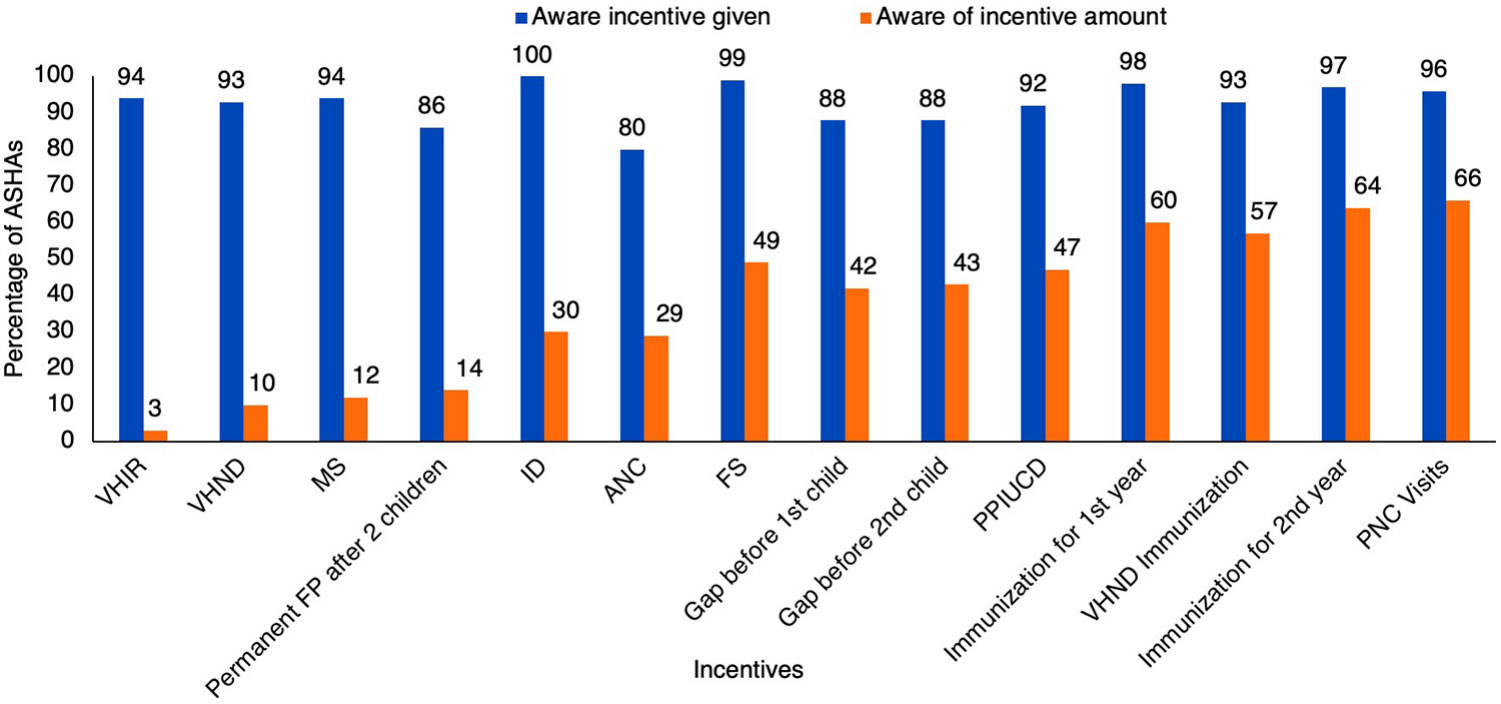
ASHA Incentive Amount Awareness Is Low^a^ Abbreviations: ANC, antenatal care; ASHA, accredited social health activist; FP, family planning; FS, female sterilization; ID, institutional delivery; MS, male sterilization; PNC, postnatal care; PPIUCD, postpartum intrauterine contraceptive device; VHIR, village health index register; VHND, village health nutrition day. ^a^ Most ASHAs are aware they get an incentive across items, but very few are aware of the exact amount; 68% ASHAs recognize the incentive amount for ID to be Indian rupee (INR) 600 and not the correct INR 300. Data from linked ASHA survey (N=1,502).

Additional areas for improvement in the incentive system are evident in ASHA's awareness of incentive amounts and experiences receiving payments. According to 2017–2018 Government of Uttar Pradesh payment data, ASHAs on average earned INR 2,580 (US$36) per month from incentives. Of the total earnings, 36% came from routine activities (INR 941 [US$13]), 31% from ANC and institutional delivery (INR 789 [US$11]), 15% from newborn and child health (INR 376 [US$5]), 15% from routine immunization (INR 387 [US$5]), and 3% from FP. While most ASHAs were aware that they could get an incentive across the activities, few knew the exact incentive amounts (Figure 3). Although 100% of ASHAs were aware of the incentive for facility delivery, only 30% knew the exact amount.

**FIGURE 4:**
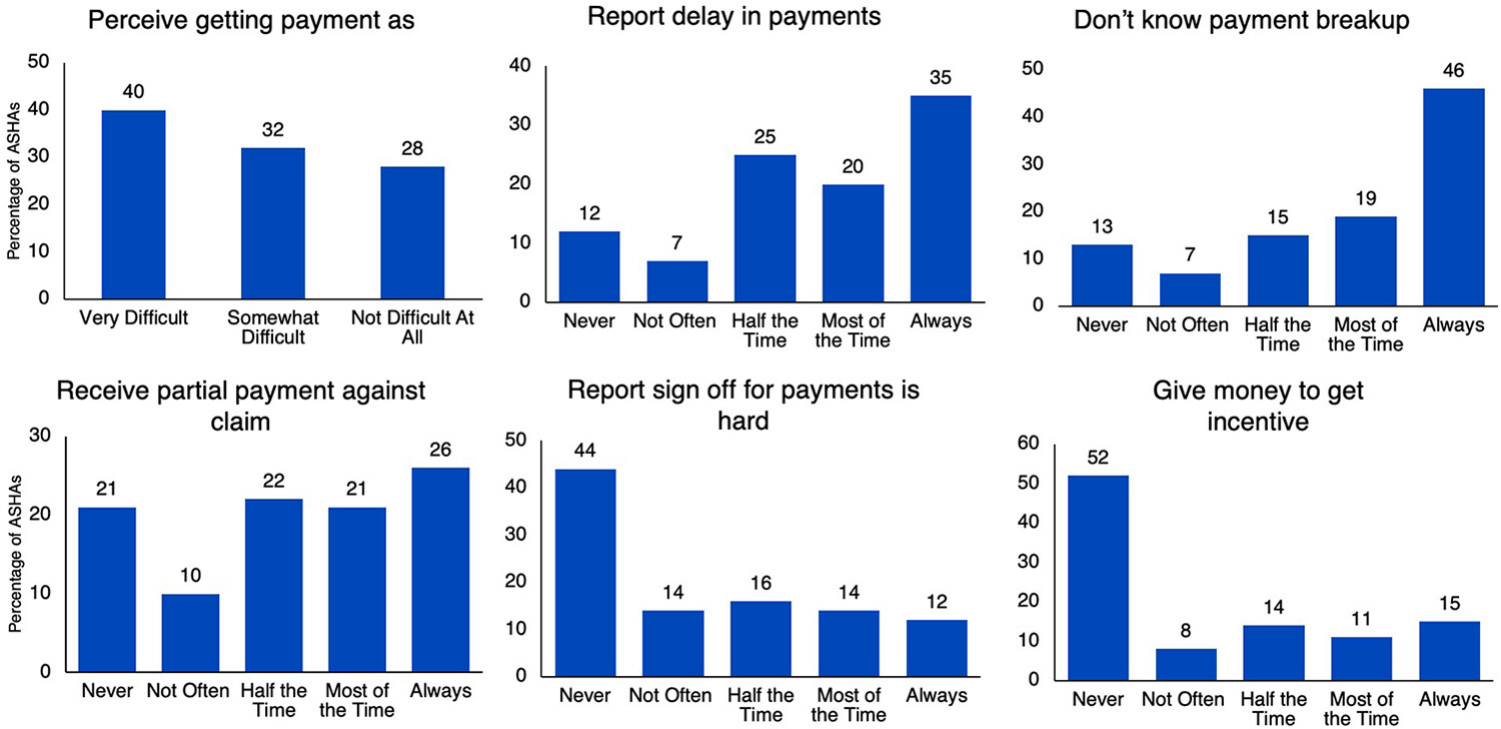
ASHA Incentive Claim Experiences (N=1,502)

At the time of the survey, 34% of ASHAs reported receiving their last incentive payment in the previous month, 31% reported receiving it 2 months before, and the remaining 35% even earlier. In addition, 55% of ASHAs received their most recent payment for 2 or more months' worth of work. ASHAs also reported suboptimal experiences claiming their incentives: 72% of ASHAs perceived getting payment as “difficult,” and 55% reported experiencing delays in payments always or most of the time. Other challenges that ASHAs reported include: 65% not knowing the payment breakup by incentive always or most of the time, 47% receiving only partial payment for their claim always or most of the time, 26% reporting sign off for payments was hard always or most of the time, and 26% having to pay to get incentives always or most of the time (Figure 4).

## DISCUSSION

The approaches employed and the implications of these findings, as relevant, extend beyond the ASHA program in Uttar Pradesh to the millions of CHWs across India and beyond. The analysis relied on a novel combination of linked household and CHW surveys, the collation of multiple data sources including primary surveys and claims data, and earnings projection models of payments and behaviors. By leveraging multiple previously uncombined data sets, we built simple but potent earnings projection models to demonstrate that **how** incentive systems are designed and implemented can impact their potential to both motivate ASHAs and drive desired outcomes.

Based on these findings and best practices for incentive design, we recommend 2 changes to the ASHA incentive system: (1) improve the design of the incentives model to help ASHA achieve outcomes, and (2) improve the implementation of incentive tracking and payments. We also discuss how the government of Uttar Pradesh has strengthened its current practices of the incentive payment system. While some recommendations are specific to the ASHA program, the underlying research on incentive design extends to any program using financial incentives for CHWs.

In our analysis, overpayment of ANC, PNC, and routine immunization incentives was found to be a result of incentives being paid for partial completion of a series of outcomes. Only initiating the series sufficed, suggesting that these incentives, as designed, may not be pushing ASHAs to complete the series. Further, ASHA actions are associated with increased maternal and newborn health outcomes.^27^ Government data report very low rates of regimen completion for 4+ ANC check-ups (26.4%), 6/7 PNC visits (54% receive a PNC visit within 2 days of delivery), and a suboptimal rate of full immunization (51.1%), suggesting that the complexity of the lumped incentives limits completion.^34^ When tested on incentive amount awareness, only 30% of ASHAs gave the correct answer of INR 300 for the institutional delivery incentive. Instead, 68% of ASHAs recognize the institutional delivery incentive amount to be INR 600—the amount given for completion of ANC and institutional delivery—demonstrating that ASHAs do not perceive ANC as a separate incentive requiring completion of additional actions. Alternatively, the lack of knowledge of specific incentives and payment for partial completion may suggest that ASHAs consider the incentives to be general compensation for the role rather than distinct incentives to complete specific tasks.

One potential solution is to isolate lumped incentives given for a series of outcomes to individual incentives for individual outcomes, which are progressive and weighted for high-impact activities or outcomes.^15^ Another option is to offer a small incentive for initiating the series of outcomes followed by a larger incentive for completion. Research shows that both task complexity and the size of incentives matter for performance.^5^^,^^11^^,^^14^^,^^15^^,^^36^ The associated demotivation that could arise among ASHAs from a solution to fix the overpayment issue cannot be undermined. Any change in incentive design must ensure that the loss in motivation is sufficiently offset by the possibility of increased reward. The proposed alternate incentive design could both decrease the complexity and increase reward, leading ASHAs to perceive every outcome as a separate initiative and assign adequate effort accordingly. There are many ways to make incentives easier for ASHAs to claim; separating lumped incentives to minimize task complexity attached to each incentive is one option. Given the paper-based incentive claims system, adding more incentives might create additional hurdles, but the increasing digitization of the system may simplify the addition. It is important to maintain a balance between reducing the number of incentives to claim and making incentives specific enough to drive specific behaviors and outcomes.

The proposed alternate incentive design could both decrease the complexity and increase reward, leading ASHAs to perceive every outcome as a separate initiative and assign adequate effort accordingly.

We find that FP incentives are the least claimed, despite promising the highest earnings. As currently designed, even if ASHAs fulfilled all FP activities under their control, earnings from these incentives will fall well short of maximum earnings owing to rigid behaviors at the household level. These rigid behaviors are demonstrated by the large INR 1,859 gap in earnings between the perfect ASHA–perfect household and perfect ASHA–actual household model for FP incentive earnings. Additionally, the timelines required for certain FP incentives (e.g., spacing 2 years between births) create additional barriers to claiming the incentive. Qualitative research conducted to inform the household survey design found that ASHAs do not view FP incentives as achievable.^37^ Research suggests that incentives for activities that recipients do not believe they can achieve (e.g., convincing families to adopt a permanent contraception strategy) fail to improve outcomes. We suggest simplifying incentives that are not perceived as achievable by ASHAs to include tasks they can accomplish.^13^^,^^24^ For example, FP incentives could focus on activities such as counseling, commodity distribution, and facilitation rather than necessitating couples to adopt permanent contraceptive methods or adequately space. Overall, these findings indicate that the ASHA incentive program in Uttar Pradesh can be strengthened, guided by best practices of incentive design such as those cited here.

In addition to potential incentive design changes, the findings show ways to improve how incentives are implemented to help ASHAs achieve outcomes and improve their understanding of the incentive system. Incentives are paid for partial completion of outcomes or activities. To deter this and thereby improve the likelihood of ASHAs completing all incentivized activities, we recommend improved validation of completion. Validation could come in the form of vouchers to households, a mobile app with fingerprint validation, or spot checks.

Additionally, many ASHAs found the claims process hard and reported payment delays. Evidence from parts of India and other low- and middle-income countries shows that CHWs are demotivated by delayed or partial disbursement of incentives.^4^^,^^15^ The Government of Uttar Pradesh introduced a short message service (SMS) system to transition from the paper-based claims process to digitized claims, to reduce delays in payments, make the payment process more transparent, and provide timely feedback to promote ASHAs' involvement. ASHAs submit paper-based vouchers to supervisors which are then submitted digitally. Once approved, ASHAs receive an SMS message with the breakdown of the incentive payments they will receive. Since its launch, payments have become timely, and the functionality and earnings of ASHAs have improved according to government program records.^38^ Further modifications and additions to the SMS system will allow for additional improvements to the incentive payment process. Moreover, by increasing transparency in payments, the system can help resolve some of the challenges around incentive awareness mentioned above. Future research evaluating the impact of these systemic improvements on CHW payments, actions, and health outcomes can provide valuable lessons around the efficacy of such systems.

There is also a role for nonfinancial incentives and other enabling factors. Simply changing the design and implementation of the compensation structure cannot enhance motivation if supportive supervision, adequate resources, technical know-how, family support, community respect and the like are not in place.^4^^–^^6^^,^^24^^,^^25^ To maximize the effectiveness of CHW incentive structures, policy makers should also consider how a CHW's environment impacts her ability and motivation to do her job. Ultimately, changes at multiple levels may need to be implemented. A mix of financial and nonfinancial incentives has proven to be effective in enhancing the performance of CHWs particularly when they perform multiple tasks in Kenya and other settings. Many studies including those from India's neighbor Bangladesh have reported that CHWs felt that they would feel motivated if they received nonfinancial incentives such as preferential access to loans or health care services and career advancement opportunities in addition to the financial incentives they were already receiving.^4^^,^^5^

Simply changing the design and implementation of the compensation structure cannot enhance CHW motivation if they lack supportive supervision, adequate resources, technical know-how, family support, and community respect.

We encourage CHW programs to actively collect and collate information to use the types of models employed in this analysis regularly to monitor incentive performance and take corrective actions. Model findings on higher potential earnings by increasing effort and maximum possible earnings could be innovatively integrated into mobile apps or other feedback platforms to nudge CHWs to increase effort in high-value, high-earning tasks and thereby achieve better outcomes. Rapid feedback loops comparing CHWs' actual earnings and potential earnings can help CHWs better understand their earnings and what to do to earn more.

### Limitations

The research is not without its limitations. For the earnings projection models, we relied on multiple data sets and reports, with varying strengths of quality, because different data sets focus on different health behaviors and estimate types. Datasets are also of different time periods, but we included the latest available data to match the time periods of different surveys or reports used in computing a particular value. Estimates used are mostly state-level averages given data availability, and therefore, any sub geography level variation is not presented in these results. While we have shown varying model results for different ASHA catchment sizes, we have not been able to provide confidence intervals for the model results as the computations rely on multiple data sets. The 4 modeled scenarios do not cover all possibilities of ASHA and household effort that might vary by incentive or household or ASHA. We had to assume there were no reporting errors in the government payments data for our analyses. Though the incentive payments are digital, there could be other factors contributing to reporting errors. Additionally, owing to the lack of incentive-specific data in some cases, we had to rely on proxy indicators, which slightly impact the estimates we produce. Finally, improvements and changes have been made to the ASHA program since the time of data collection, which may not be reflected in the reported results.

## CONCLUSION

Community health workers play a crucial role in global health systems. As many programs employ financial incentives to influence CHW performance, developing methods for analyzing the design of these incentives is essential for improving the impact of CHW channels. While we do not advocate a specific model of compensation, it is important to systematically study whether any given compensation model is impactful and to develop adaptive systems so that the structure of compensation can be revised based on learnings. Moreover, systems should be planned with the evidence-based principles of incentive design in mind. Modeling payments as we have done here can enable programs to devise incentive systems that effectively drive target outcomes.

## Supplementary Material

GHSP-D-21-00413-supplement.pdf

## Notes

**Peer Reviewed**
