# Supplementary material for: Improving Community Health Worker Compensation: A Case Study From India Using Quantitative Projection Modeling and Incentive Design Principles
Source: Glob Health Sci Pract. 2022 Jun 29;10(3):e2100413. doi: 10.9745/GHSP-D-21-00413 (PMC9242609; doi:10.9745/GHSP-D-21-00413)
Supplement: GHSP-D-21-00413-supplement.pdf [file GHSP-D-21-00413-supplement.pdf]

Supplement to: Jain M, Caplan Y, Ramesh BM, et al. Improving community health worker compensation: a case study from India using quantitative projection modeling and incentive design principles. *Glob Health Sci Pract*. 2022;10(3):e2100413. https://doi.org/10.9745/GHSP-D-21-00413

Supplement Table 1: RMNCH Incentives and data sources

Incentive	Amount (in ₹)	Data source	Geography
For completion of all components of antenatal care (3+ ANC checkups, administration of 2 TT shots, Hemoglobin and Urine examination, 100 Iron Folic Acid consumption)	300	Surgo-UPTSU RMNCH survey with households and ASHAs (2017), SRS - CBR (2017)	Statewide
For completion of institutional delivery, verified by hospital discharge slip	300	Surgo-UPTSU RMNCH survey with households and ASHAs (2017), SRS - CBR (2017)	Statewide
For completion of 6 postnatal home visits for institutional deliveries and 7 for home births	250	Surgo-UPTSU RMNCH survey with households and ASHAs (2017), SRS - CBR (2017), SRS - NMR (2016)	Statewide
For House Visit on return of malnourished children from Nutrition Rehabilitation Centre or after Community Based Malnutrition Management	150	Census (2011; projected for 2016), RSOC (2013-14)	25 High Priority Districts (HPDs)
For two House Visits every month on return of underweight children from Sick Newborn Care Unit (SNCU) (Till two years)	50	SRS - CBR (2017), UPTSU Case Sheet Summary (2016), CBTS 3-G1 (2016)	25 HPDs
For monthly House Visits on return of normal weight children from SNCU (Till two years)	50	SRS - CBR (2017), UPTSU Case Sheet Summary (2016),	25 HPDs

		CBTS 3-G1 (2016)	
For encouraging beneficiaries to go to Village Health and Nutrition Day and attend it	200	Surgo-UPTSU RMNCH survey with households and ASHAs (2017)	Statewide
For encouraging people to bring children on Vaccination Day	150	Surgo-UPTSU RMNCH survey with households and ASHAs (2017)	Statewide
For complete immunization of children in the first year	100	SRS - CBR (2017), Surgo-UPTSU RMNCH survey with households and ASHAs (2017), NFHS-4 State Factsheet for UP (2015-16)	Statewide
For complete immunization of children in the second year	50	SRS - CBR (2017), Surgo-UPTSU RMNCH survey with households and ASHAs (2017), NFHS-4 State Factsheet for UP (2015-16)	Statewide
For helping woman get tubectomy	200	Government of India Order (2012), UPTSU DLFPS (2016)	25 HPDs
For helping man get sterilized	300	Government of India Order	25 HPDs

		(2012), UPTSU DLFPS (2016)	
For encouraging couples to go for permanent contraception method after two children	1,000	Government of India Order (2012), UPTSU DLFPS (2016)	25 HPDs
For encouraging couples to keep a gap of 2 years for first child after marriage	500	Government of India Order (2012), UPTSU DLFPS (2016)	25 HPDs
For encouraging couples to keep a gap of 3 years between the first and the second child	500	Government of India Order (2012), UPTSU DLFPS (2016)	25 HPDs
For encouraging couples to get PPIUCD after childbirth and for taking to hospital	150	Surgo-UPTSU RMNCH survey with households and ASHAs (2017), SRS - CBR (2017)	Statewide
For making list of all houses at start of year and updating it every six months	100	Surgo-UPTSU RMNCH survey with households and ASHAs (2017)	Statewide
For making Village Health and Index Register and for registration of all birth and death	100	Surgo-UPTSU RMNCH survey with households and ASHAs (2017)	Statewide
For making a list of beneficiaries for vaccination and updating it every month	100	Surgo-UPTSU RMNCH survey with households and ASHAs (2017)	Statewide

Supplement to: Jain M, Caplan Y, Ramesh BM, et al. Improving community health worker compensation: a case study from India using quantitative projection modeling and incentive design principles. *Glob Health Sci Pract*. 2022;10(3):e2100413. https://doi.org/10.9745/GHSP-D-21-00413

For making a list of beneficiaries of antenatal examination and updating it every month	100	Surgo-UPTSU RMNCH survey with households and ASHAs (2017)	Statewide
For making a list of suitable couples and updating it every month	100	Surgo-UPTSU RMNCH survey with households and ASHAs (2017)	Statewide
For organizing meeting of Committee on Village Health, Cleanliness and Nutrition	150	UPTSU Program Monitoring (2015-16)	100 TSU blocks
For attending meeting at Block Primary/Community Healthcare Centre	150	UPTSU Program Monitoring (2015-16)	100 TSU blocks

Supplement to: Jain M, Caplan Y, Ramesh BM, et al. Improving community health worker compensation: a case study from India using quantitative projection modeling and incentive design principles. *Glob Health Sci Pract*. 2022;10(3):e2100413. https://doi.org/10.9745/GHSP-D-21-00413

Supplement Table 2: ASHA Incentives

Category	Incentive/ Activity	Incentive Amount (INR)
**Janani Suraksha Yojana**	For antenatal care	300
	Promoting institutional delivery	300
**Death Reporting**	Maternal death reporting	200
**Referral**	Refer High Risk Pregnant woman to higher center for further diagnosis, treatment and institutional delivery and support in entering the detail in Mother and Child Tracking System/Reproductive and Child Health portal	300
**Home Based Newborn Care**	Newborn care home visits under Home Based Newborn Care Program for module 6-7 trained ASHAs	250
**Nutrition Rehabilitation Centre**	For House Visit on return of malnourished children from Nutrition Rehabilitation Centre or after Community Based Malnutrition Management (Till girth of mid portion of upper arm becomes 125 mm)	150
**Sick Newborn Care Unit**	For two House Visits every month on return of underweight children from SNCU (Till two years)	50
	For monthly House Visits on return of normal weight children from SNCU (Till two years)	50
	Referring Severe Acute Malnourished child to Nutrition Rehabilitation Center	50
	4 follow-ups of children after discharge from NRC (Per Child)	100
**Village Health & Nutrition Day**	For encouraging beneficiaries to go to Village Health and Nutrition Day and attend it	200
**Regular Vaccination Program**	Mobilizing Children at VHND for immunization (Per Session)	150
	For complete immunization of children in the first year	100
	For complete immunization of children in the second year	50
**Family Welfare**	Female Sterilisation	200
	Male Sterilisation	300
	For encouraging couples to go for permanent contraception method after two children	1,000
	For encouraging couples to keep a gap of 2 years for first child after marriage	500
	For encouraging couples to keep a gap of 3 years between the first and the second child	500

**Family Welfare (Safe Abortion services)**	For encouraging couples to get PPIUCD after childbirth and for taking to hospital	150
	Motivating and mobilizing beneficiary	150
	Mobilizing woman to hospital for safe abortion services through surgical method	150
	Mobilizing woman to hospital for safe abortion services through medical method and 3 follow-ups	225
**Revised National Tuberculosis Control Programme**	On completion of treatment of new patients of T.B. (Category-1)	1,000
	On completion of treatment of new patients of T.B. (Category-2)	1,500
	For completion of treatment of MDR patients (IP-₹ 2000 CP-₹ 3000)	5,000
**Leprosy**	Identification of leprosy patients	250
	Pauci Bacillary (Complete treatment)	400
	Multi Bacillary (Complete treatment)	600
**Malaria**	Blood Platelets or Rapid Diagnostic test	15
	On getting complete treatment for confirmed case of Malaria	75
**Black Fever**	For identification, referral and getting complete treatment of Black Fever patients	300
**Lymphatic Filariasis**	For making list of all lymphatic and hydrocele cases	200
	For surveying 50 houses or 250 people every day (up to maximum 3 days)	200
**Encephalitis**	For referral of A.E.S./J.E. case to nearest Community Healthcare Centre/District Hospital/Medical College	300
**Village Health Index Register**	For making list of all houses at start of year and updating it every six months	100
	For making Village Health and Index Register and for registration of all births and deaths	100
	For making a list of beneficiaries for vaccination and updating it every month	100
	For making a list of beneficiaries of antenatal examination and updating it every month	100
	For making a list of suitable couples and updating it every month	100
**For meeting of Committee on Village Health, Cleanliness and Nutrition**	For organizing meeting of Committee on Village Health, Cleanliness and Nutrition	150

**For Primary Health Centre meeting**	For attending meeting at Block Primary/Community Healthcare Centre	150
**Rashtriya Kishor Swasthya Karyakram**	Peer Educator	50
	Motivating Adolescents to attend Adolescent health day	200

Supplement Table 3: Model Outputs for Varying ASHA Catchment Sizes

Catchment size (population)	P-P Model	P-A Model	A-P Model	A-A Model
**1000**	**INR 5867**	**INR 3000**	**INR 2009**	**INR 1325**
500	INR 3509	INR 2075	INR 1421	INR 1079
750	INR 4688	INR 2538	INR 1715	INR 1203
1250	INR 7047	INR 3463	INR 2303	INR 1449
1500	INR 8226	INR 3925	INR 2596	INR 1572

Supplement to: Jain M, Caplan Y, Ramesh BM, et al. Improving community health worker compensation: a case study from India using quantitative projection modeling and incentive design principles. *Glob Health Sci Pract*. 2022;10(3):e2100413. https://doi.org/10.9745/GHSP-D-21-00413

Supplement Methods

Surgo-UPTSU RMNCH survey with households and ASHAs

Households: recently delivered women (n=5,469), their husbands (n=3,064), mothers-in-law (n=3,626) were interviewed on their practices, knowledge, beliefs and perceptions of pregnancy, health behaviors, interactions with and perceptions of the ASHA and other health system components, personality attributes, household dynamics and sociodemographics. ASHAs (n=1052) were surveyed on their actions, knowledge, beliefs and perceptions around different health behaviors, interaction with and perception of their communities, supervisors, health system, job attributes, and sociodemographics. ASHAs were also asked about their incentive earnings and incentive system knowledge. The ASHA survey included an audit and review of their record keeping, both for a log of their annual beneficiaries and to audit their record keeping as several incentives are given to ASHAs for monthly record keeping. We applied correction sample weights to generate state level estimates.

This dataset served as the primary source for calculating beneficiary incidence (live birth rate), ASHA action (ASHA encouraged woman to go for ANC check-ups, ASHA encouraged woman to go for facility delivery, ASHA conducted PNC home visits, ASHA encouraged woman to go for child immunization, ASHA attended VHND with at least one woman, ASHA counselled on post-partum FP, ASHAs conducted routine activities) and household behavior rates (woman completed full ANC, woman delivered at a facility, woman adopts PPIUCD) needed to model ASHA incentive claims.

UPTSU District Level Family Planning Survey

High priority districts (HPD) were determined by the government based on indicators of the Maternal Mortality Ratio (MMR), % of safe deliveries, Infant Mortality Rate (IMR), % of children 12-23 months fully immunized, Total Fertility Rate (TFR), and Contraceptive Prevalence Rate (CPR) – Modern Method. Sample size was based on prevalence of modern contraceptive users in the Annual Health Survey 2012-2013. The survey used a two-stage cluster sampling design. First five blocks were selected in each district in proportion to population size. Second, ASHA areas were used as sampling units for random selection. The number of ASHA areas in each district was decided considering a total of five interviews could be conducted. After random selection, five women were interviewed in each PSU by a female investigator between April and August 2016. A final sample of 13,182 married women was achieved. Survey analysis applied necessary sampling weights to produce robust estimates at the 25 HPD level. The limitation of this data set is that statewide estimates are not available.

UPTSU Community Behavior Tracking Survey (CBTS)

UPTSU designed a set of rolling periodic population-based surveys called Community Behavior Tracking Surveys (CBTS) to generate evidence on coverage and utilization of health services as well as health outcomes. These surveys considered the health units (subcentre, ASHA area etc.)

in sampling design to improve the utility of the data by block/district program managers. It is intended to generate information on short-term changes and to gather near real-time data required for program monitoring, which are not readily available from most other surveys as they cover broad population groups (ever-married/currently married women age 15-49) and births in the past 3 or 5 years. Therefore, CBTS, as a short survey was implemented more frequently, and was better focused to track short-term changes in indicators at smaller geographic areas. The CBTS includes four demographic groups: women who ended their pregnancy in the last 2 months, mothers with children age 3-5 months, mothers with children age 6-11 months and mothers with children age 12-23 months. The CBTS (2016) study followed a three-stage sampling design and was conducted across 25 HPDs in UP to provide district level estimates of key program indicators. Required number of samples for each HPD was estimated based on district specific prevalence of key RMNCH indicators from the Annual Health Survey, 2012-13. In the first stage, 5 blocks from each HPD were selected using Probability Proportional to Size (PPS) method. Within each block, the number of primary sampling units (PSUs) were estimated using simple random sampling approach. Considering the smallest service delivery unit of ASHA areas as the primary sampling units, the study selected 2,697 ASHAs randomly and selected all the eligible women (who satisfy the criteria of the four survey groups) for the interview. The limitation of this data set is that statewide estimates are not available.

UPTSU Case Sheet Summary Data

The UPTSU case sheet summary data summarizes delivery related clinical data from the job aid tool designed for nurses to manage deliveries and complications. The data is available for 150 blocks in the 25 HPDs of Uttar Pradesh. The limitations of this data set are that statewide estimates are not available, and this dataset might be prone to data quality issues around standardization given the data comes from a job aid tool that nurses use to manage their work.

UPTSU Program Monitoring Data

The UPTSU program monitoring data summarizes information on 5 key indicators (pregnancy registration, ANC check-up, institutional delivery, PNC home visits, and use of modern contraceptive) collected from ASHAs that visit the monthly block level meetings. The data is available for 100 blocks in the 25 HPDs of Uttar Pradesh. The objective of the dataset is to record and identify critical gaps in community interventions led by ASHAs. The limitations of this data set are that statewide estimates are not available, and this dataset might be prone to data quality issues given the data comes from a job aid tool that community health workers use to manage their work.
